# An Integrated Autonomous Dynamic Navigation Approach toward a Composite Air–Ground Risk Construction Scenario

**DOI:** 10.3390/s24010221

**Published:** 2023-12-30

**Authors:** Da Jiang, Meijing Wang, Xiaole Chen, Hongchao Zhang, Kang Wang, Chengchi Li, Shuhui Li, Ling Du

**Affiliations:** 1 National Engineering Laboratory for Wheeled Vehicle, China North Vehicle Research Institute, Beijing 100072, China; ziangdar@sina.com (D.J.); 15201615492@163.com (M.W.); d202187005@hust.edu.cn (H.Z.); dreamy_on2023@163.com (S.L.); duling0615@163.com (L.D.); 2 School of Mechanical Science and Engineering, Huazhong University of Science and Technology, Wuhan 430074, China; m202170779@hust.edu.cn (K.W.); d202280230@hust.edu.cn (C.L.)

**Keywords:** motion planning, hierarchical decision, self-adaptive dynamic window approach, risk assessment

## Abstract

Unmanned transportation in construction scenarios presents a significant challenge due to the presence of complex dynamic on-ground obstacles and potential airborne falling objects. Consequently, the typical methodology for composite air–ground risk avoidance in construction scenarios holds enormous importance. In this paper, an integrated potential-field-based risk assessment approach is proposed to evaluate the threat severity of the environmental obstacles. Meanwhile, the self-adaptive dynamic window approach is suggested to manage the real-time motion planning solution for air–ground risks. By designing the multi-objective velocity sample window, we constrain the vehicle’s speed planning instructions within reasonable limits. Combined with a hierarchical decision-making mechanism, this approach achieves effective obstacle avoidance with multiple drive modes. Simulation results demonstrate that, in comparison with the traditional dynamic window approach, the proposed method offers enhanced stability and efficiency in risk avoidance, underlining its notable safety and effectiveness.

## 1. Introduction

Real-time motion planning holds significant importance within the realm of autonomous driving applications. Extensive research has been conducted on motion planning in structured environments for unmanned ground vehicles (UGVs) [1,2,3,4,5,6]. However, quantity research focuses on general ground risk-avoidance motion planning, overemphasizing the target identification or kinematic-level reactive maneuvers, while downplaying the vehicle’s dynamics-level planning constraints. Moreover, it overlooks the need for reactive evasive maneuvers in response to abrupt intrusions by airborne threats. The issue is particularly pronounced in the navigation problem of unmanned transport vehicles (UTVs) in construction scenarios. On the one hand, the control system needs to balance vehicle dynamics constraints and the rapid dynamic obstacle avoidance requirements online. On the other hand, the UTV needs to swiftly perform evasive maneuvers in extreme response times to mitigate high-speed falling objects while ensuring compliance with vehicle dynamic capabilities.

This article aims to design an auxiliary motion function module for motion planning in construction scenarios with coupled mobility risks. Once an aerial threat is detected, the module is promptly activated to real-time plan the reactive maneuver trajectory for the vehicle within an extremely short response time, aiming to maximize vehicle safety toward the rapid descent hazards.

Despite efforts in some research to quantify airborne threats as either general ground risks or ground risks with altitude information [7,8], practical applications of UTVs reveal that spatial threats cannot be adequately characterized as typical ground threats. This distinction is especially pronounced in construction scenarios. Firstly, within the maneuvering operation time scale, not all detected obstacles need to be classified as potential risks. For instance, in construction scenarios, a multitude of ground obstacles such as people, vehicles, buildings, and construction materials are present, whereas only targets closely positioned in the expected trajectory’s spatial proximity will significantly impact or pose a threat to the planning trajectory. Furthermore, airborne objects like falling leaves and small balloons, although detected as airborne targets in opposing motion, do not pose risks to the travel of UTVs. Therefore, through isolating or discounting redundant low-risk factors, risk quantification assessment for the various detected targets needs to be employed to reduce planning costs within the short-term planning window.

The anticipated on-ground distance metric generally has a positive effect during the ground risk assessment. However, this metric should not be directly migrated to the risk assessment process for airborne objects, as the vehicle’s altitude would affect the risk assessment [9,10,11,12]. For instance, once an airborne object approaches UTVs on a horizon or slightly inclined trajectory, its anticipated landing spot would be at a significant distance from the vehicle. Thus, the original metric would deem this trajectory as safe, while the collision would indeed occur. In addition, the presence of airborne objects results in an extremely short maneuvering time scale for UTVs (on the order of seconds). This greatly emphasizes the importance of risk quantification assessment in filtering objects considered redundant. Moreover, the short time scale for risk avoidance operations could frequently lead to vehicles employing aggressive control maneuvers, imposing significant dynamic burden or danger, such as the heightened risk of rollovers during a high-speed turning maneuver. So, it is essential to integrate the dynamics constraints into the planning. Above all these considerations, it is necessary to conduct reasonable risk quantification assessments for both ground and airborne obstacles, to streamline redundant obstacles by maintaining the planning effectiveness of the UTV, and eventually to reduce the computation costs of large-scale multi-object motion planning in stereospace.

Our contribution to this study is offering a hierarchical self-adaptive motion planning method for UTVs in construction scenarios, which successfully addresses the integrated air–ground risk avoidance problem. This work provides an efficient planning solution for the coupled risk field in the construction scene. The organization of this paper is as follows: Section 2 describes our proposed planning scheme and UTVs’ dynamics constraint model for the construction scenario. Section 3 explains the theoretical methodology of the hierarchical decision and self-adaptive planning method in detail. In Section 4, simulations are conducted and the result is presented and discussed. Finally, the conclusion and future work are addressed in Section 5.

## 2. Related Works

Local planning for unmanned vehicles is a crucial component of autonomous driving systems, aiming to enable vehicles to navigate safely and efficiently in complex and dynamic environments. In recent years, researchers have focused on addressing the challenge of coupled air–ground obstacle avoidance to enhance the reliability and adaptability of unmanned vehicles in complex scenarios.

There is existing prior research that independently analyzes the dynamic obstacle avoidance for airborne risk. Chen et al. [13] proposed an A*-cubic-spline-based dynamic planning method for an unmanned vehicle under sudden threats. Once a sudden threat is encountered, the corresponding parameters are set according to the cubic spline second-order continuity, and multiple candidate trajectories are generated. Then, the optimal trajectory is obtained according to the set objective function. This method does not meet the time margin for abrupt intrusions by airborne threats. Feng et al. [14] proposed a new dynamic path planning algorithm based on the modified artificial potential field algorithm. Once an obstacle enters the observation range of the vehicle, the movement information of the obstacle will be observed and recorded in the obstacle grid map. The prediction of obstacle position is obtained with the trajectory evaluation algorithm based on the Markov chain. Zhou et al. [15] proposed a bio-inspired path planning algorithm, which utilized the A* search algorithm to explore the generated probability map, and designed an artificial-field-based objective function, to obtain an original optimal collision-free path. The computational cost of this coupling algorithm is also relatively high. Recent decades have witnessed that deep reinforcement learning provides new perspectives for optimizing control problems in unmanned vehicles [16,17]. Singla et al. [18] proposed a deep reinforcement learning planning method based on a recurrent neural network and temporal attention. This method enables the UAV controller to collect, and store relevant observations gathered over time and use them to make better obstacle avoidance decisions. Kulathunga et al. [19] proposed a hybrid approach that combines a Montecarlo tree search and an RL-based approach to solve the 3D path planning problem. The end-to-end data-driven method is primarily tailored to specific vehicle models, exhibiting a high dependence on environmental modeling and limited generalization capability.

However, these approaches have not considered the real-time dynamic limits of the vehicle, and require calculations for all aerial obstacles, incurring high computational costs. Hence, these approaches are not suitable for a construction scenario with high maneuverability demands. In summary, researchers have conducted in-depth studies on path planning and control methods to address the challenge of the coupled air–ground obstacle avoidance problem. These efforts provide important theoretical foundations and practical experiences for achieving safe and efficient local path planning for unmanned vehicles in complex environments.

## 3. Problem Statements

**Figure 1:**
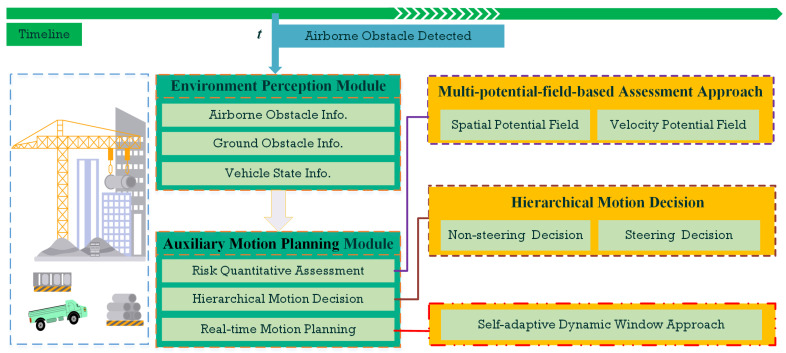
Conceptual overall sketch for the proposed motion planning method.

The conceptual sketch illustrating the motion planning mechanism process for the construction navigation scenario is presented in Figure 1. During the UTV executing the transportation mission at the construction site, the environment perception system continuously detects and identifies surrounding obstacles, including on-ground and airborne objects. Under normal circumstances, the vehicle primarily engages in real-time navigation and obstacle avoidance within ground-level environments. Once the perception system perceives descending airborne obstacles, the planning system would be swiftly handed over to the auxiliary motion planning module specialized in addressing aerial-to-ground obstacle scenarios. This module performs the entire planning and decision-making process, encompassing risk quantitative assessment, hierarchical motion decision, and real-time evasion planning function.

Consequently, we can quantify specialized spatial obstacles into general 3D obstacles with the constraints of planning time scale, vehicle’s rapid maneuvering capabilities and security logic, etc. Thus, the UTV emergency planning problem with aerial–ground risks in construction scenarios can be transformed into a dynamic closed-loop optimization problem:

(1) $$\begin{array}{c} \min:J\\ s.t.\;\Phi_{t}\leq 0\;\Phi_{m}\leq 0\;\Phi_{s}\leq 0 \end{array}$$

where $\Phi_{t}$, $\Phi_{m}$, and $\Phi_{s}$ denote the constrain items of planning time scale, maneuvering capabilities, and security logic, respectively. J denotes the objective function value of the planning optimization problem. In this paper, the detail objective function is defined as:

(2) $$J(v,\omega )=-\sigma (\mu \cdot heading(v,\omega )+\beta \cdot dist_{g}(v,\omega )+\eta \cdot dist_{o}(v,\omega )+\gamma \cdot vel(v,\omega ))$$

where heading, $dist_{g}$, $dist_{o}$, and vel denote the objective terms related to the target heading, target distance, obstacle distance, and velocity performance, respectively. σ, μ, β, η, and γ denote the corresponding coefficients, respectively.

### 3.1. Vehicle Prediction Model

**Figure 2:**
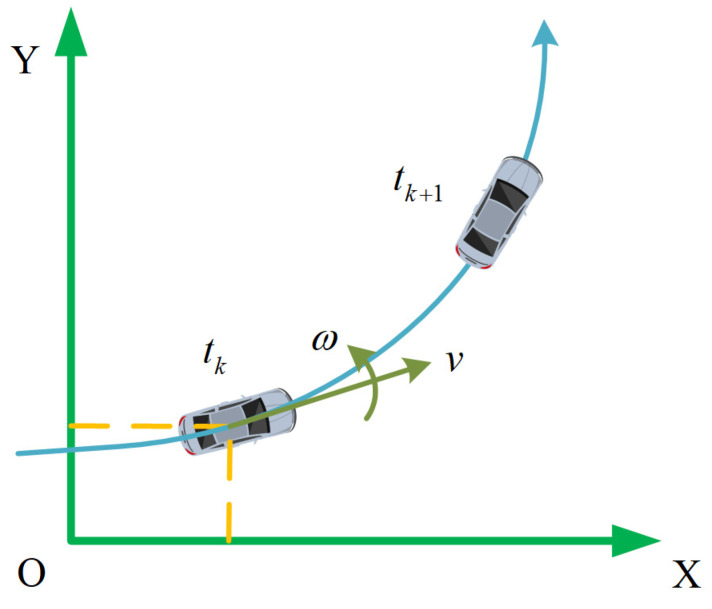
CTRV prediction model sketch.

During the actual traveling process, it is challenging to obtain real-time acceleration information from the vehicle due to the variation fluctuations and interference noise. Since speed metrics are relatively easier to obtain and have lower interference noise, to facilitate UTV’s trajectory prediction analysis, this paper employs the constant turn rate and velocity (CTRV) model to describe the UTV’s motion mechanism in the planning problem. In the CTRV prediction model, the vehicle’s linear velocity and steering rate in each time step are considered constant, as shown in Figure 2.

### 3.2. Risk Quantitative Assessment

**Figure 3:**
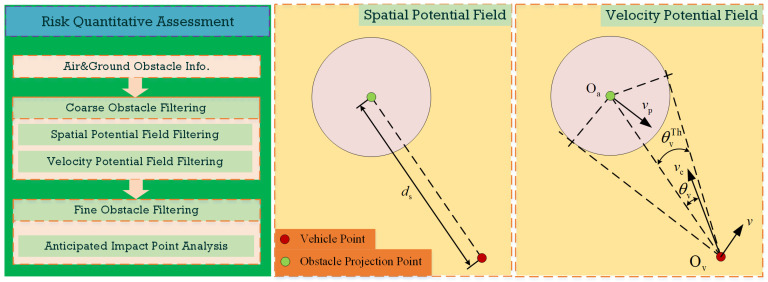
Risk quantitative assessment sketch.

Building upon the adopted CTRV vehicle prediction model, the primary illustration of the risk quantitative assessment sketch for an air–ground obstacle is presented in Figure 3. The assessment system conducts real-time calculations based on incoming air–ground obstacle data. It employs a systematic coarse filtering method to reduce redundant risk points. Following this initial screening, the system applies an additional approach to analyze potential risks associated with airborne obstacles by pinpointing their expected impact points on the vehicle. This risk assessment process is continuously executed at each sampling time step in parallel. Therefore, the high-risk obstacles are seamlessly incorporated into the real-time motion planning problem.

The spatial potential field model is employed to assess both ground and airborne obstacles simultaneously. It is achieved by analyzing whether the anticipated relative distance $d_{s}$ is projected to reach the danger distance threshold $d_{s}^{\mathrm{Th}}$. If the threshold is exceeded, the assessment system would hold that the target in question poses a threat to the vehicle at that future moment. For instance, when dealing with airborne obstacles, the system would detect the corresponding trajectory sequence points. Building upon this, the anticipated landing time and landing coordinates of the airborne obstacle could be calculated. Thus, by comparing the calculated distance $d_{s}$ from the anticipated vehicle to the landing coordinates with the predetermined threshold $d_{s}^{\mathrm{Th}}$, the system achieves a spatial-potential-field-based risk assessment. The corresponding assessment Boolean indicator $R_{s}$ can be denoted as:

(3) $$R_{s}=\left\{\begin{array}{cc} \mathrm{True} & d_{s}<d_{s}^{\mathrm{Th}}\\ \mathrm{False} & d_{s}>d_{s}^{\mathrm{Th}} \end{array}\right.$$

Once an airborne obstacle approaches the vehicle in a horizontal or low-angle direction, the obstacle’s anticipated landing point would be distant from the vehicle’s anticipated position. In such a case, the spatial potential field model is not applicable to the obstacle impacting a vehicle body scene. To address this situation, the velocity potential field model is introduced to supplement the evaluation of the risk posed by horizontal/low-angle-approaching airborne obstacles. This model assesses the anticipated risk by considering the angle between the current obstacle–vehicle composite velocity vector $v_{c}$ and the obstacle direction vector $\vec{O_{v}O_{a}}$. For instance, assuming the current translational velocity of the vehicle and airborne obstacle as v and $v_{p}$, respectively, we can obtain the composite velocity vector $v_{c}=v-v_{p}$. When the angle $\theta_{v}$ between v and $v_{p}$ falls within the corresponding expansion angle $\theta_{v}^{\mathrm{Th}}$ in the velocity potential field, the system identifies this airborne obstacle as a danger. The corresponding assessment Boolean indicator $R_{v}$ can be denoted as:

(4) $$R_{v}=\left\{\begin{array}{cc} \mathrm{True} & \theta_{v}<\theta_{v}^{\mathrm{Th}}\\ \mathrm{False} & \theta_{v}>\theta_{v}^{\mathrm{Th}} \end{array}\right.$$

For the airborne obstacles identified as $R_{v}=\mathrm{True}$ during the assessment, an additional refined risk assessment is performed through the anticipated impact point analysis for the vehicle. The coordinates of the incoming obstacle’s trajectory consequence points are transformed into the coordinates under the vehicle’s reference frame, denoted as PIm. If PIm falls outside the threshold range of the vehicle’s bounding box, it indicates that the incoming obstacle would not collide with the vehicle body. While if PIm falls within the threshold range of the vehicle’s bounding box, it signifies that the incoming obstacle is on a collision course with the vehicle at that moment. Then, the risk assessment system would output the coordinates of the impact point and the corresponding timestamp.

At this stage, based on the spatial/velocity potential field and anticipated impact point analysis, the risk assessment module could real-time filter out obstacle data that pose a genuine threat indeed. These filtered data serve as the basis for subsequent real-time motion planning assessments.

## 4. Methodology

**Figure 4:**
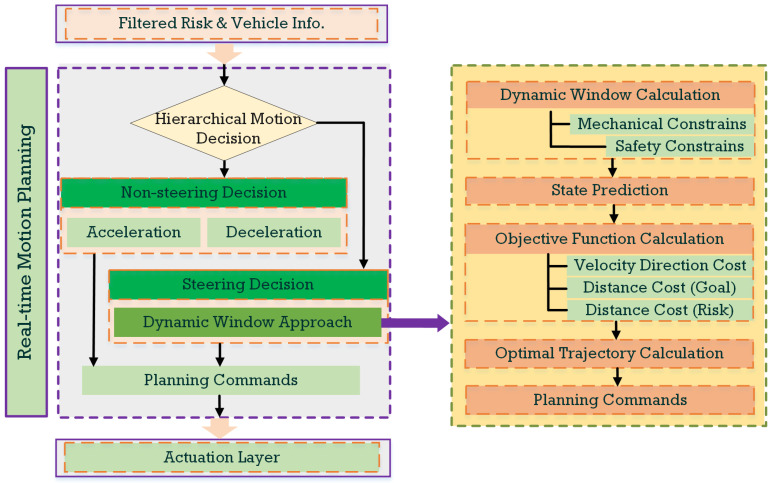
The proposed local motion planning framework.

In intricate construction environments with complex air–ground risks, the restricted maneuvering time scale for UTVs necessitates swift obstacle avoidance, corresponding to a local motion planning issue. Thus, this paper introduces a real-time self-adaptive local motion planning approach based on the dynamic window approach (DWA). This method is employed to resolve dynamic optimization problems posed by the construction scenario motion planning. The local motion planning framework presented in this paper is depicted in Figure 4.

### 4.1. Hierarchical Motion Decision

The primary distinction between airborne and ground obstacles centers on the temporal dimension. Airborne threats are inherently time-dependent, existing as potential risks only within a short time scale from the moment of detection until they land on the ground. As a result, the hierarchical motion decision approach, combining the non-steering avoidance decision and steering avoidance decision, is deemed effective for managing airborne obstacles.

Differing from typical hierarchical decision approaches, which are based on temporal or informational flow [20,21], the proposed non-steering/steering-based hierarchical approach in this paper aims to decrease the destabilizing risks associated with rapid steering actions. Non-steering avoidance involves trajectory adjustments through pure acceleration or deceleration without altering the vehicle’s original path, primarily focusing on longitudinal obstacle avoidance. Conversely, steering avoidance employs the vehicle’s steering mechanism to execute avoidance maneuvers that account for both lateral and longitudinal aspects of its movement.

Thus, the hierarchical motion decision, encompassing both non-steering and steering avoidance is deployed. Notably, rapid and sharp steering maneuvers, especially at high speed, pose a certain risk to vehicle stability, potentially resulting in skidding or rollovers and, consequently, intensifying the risk. The steering avoidance method, which considers rollover prevention mechanisms, necessitates additional iterations within the dynamic window search, leading to lengthier computational processes and placing greater demands on the control system. Conversely, non-steering avoidance through acceleration and deceleration provides smoother vehicle control, mitigates the risk of instability, and allows the control system a higher response time. Consequently, in emergency scenarios, non-steering approaches are typically given precedence. Within each time window, the vehicle first assesses the feasibility of applying non-steering avoidance strategies. If a viable non-steering solution cannot be generated, steering avoidance strategies are then considered.

### 4.2. Dynamic-Window-Approach-Based Real-Time Planning Method in Construction Scenario

With the insights of the anticipated impact point, the 3D air–ground obstacle avoidance problem can be converted into a 2D problem. The proposed self-adaptive dynamic window approach (ADWA) for steering-based local motion planning in construction scenarios is illustrated in Algorithm 1. In contrast to the traditional DWA [22], the ADWA introduces an adaptive prediction horizon mechanism that tunes the length of the prediction horizon based on obstacle information. This mechanism effectively mitigates the issues encountered in DWA, where a fixed prediction horizon can result in insolvable scenarios in dense obstacle-laden environments. In contrast, the adaptive dynamic window approach adjusts the prediction horizon length by considering the number of nearby obstacles, providing a more flexible response to complex environments.

Theoretically, the ADWA excels in its adaptability to the dynamic nature of the environment. As obstacle density increases, the adaptive dynamic window automatically reduces the prediction horizon length to respond more sensitively to potential collision risks. Conversely, in relatively open environments, the prediction horizon length can be moderately increased to enhance planning efficiency. This flexibility makes the adaptive dynamic window approach more suitable for a variety of real-world scenarios, balancing path planning safety and efficiency in narrow passages as well as open areas. In summary, the adaptive dynamic window approach overcomes the limitations of traditional dynamic window methods in dealing with complex environments by dynamically adjusting the prediction horizon length during the planning process, thereby improving the adaptability and robustness of the planned trajectory.

Furthermore, during the calculation of admissible velocities within the dynamic window, ADWA takes into account critical physical constraints, including the maximum mechanical steering angle and critical rollover velocity. This ensures a more practical and realistic planning approach in rapid maneuvering scenarios.

During the optimization process outlined in Algorithm 1, the vehicle’s dynamic window is influenced by various factors, including speed limits, acceleration constraints, and obstacle avoidance requirements. The corresponding sampled spaces are as follows:

(5) $$\begin{array}{l} V_{s}=\left\{(v,\omega )\left||v|\leq v_{\max}\cap |\omega |\leq \omega_{\max}\right.\right\}\\ V_{d}=\left\{(v,\omega )\left|v\in [v_{0}-a_{\max}\Delta t,v_{0}+a_{\max}\Delta t]\cap \omega \in [\omega_{0}-\alpha_{\max}\Delta t,\omega_{0}+\alpha_{\max}\Delta t]\right.\right\}\\ V_{a}=\left\{(v,\omega )\left||v|\leq \sqrt{2dist(v,\omega )a_{\max}}\right.\right\} \end{array}$$

where $v_{\max}$, $\omega_{\max}$, $a_{\max}$, and $\alpha_{\max}$ denotes the maximum for the vehicle’s translational velocity, rotational velocity (angle velocity), translational acceleration, and rotational acceleration, respectively.
**Algorithm 1. Self-adaptive dynamic window approach**1. Exploration domain: The exploration domain of the dynamic window can be restricted by: (a) Circular trajectories: The possible trajectories of the ADWA can be described by the velocity pairs (v,ω). (b) Restricted velocities: Vehicles need to leave sufficient braking distance for each obstacle; hence, the velocity pairs (v,ω) need to be restricted. (c) Dynamic window: The dynamic performance of the vehicle constrains the variation range of the dynamic window at each sampling moment. (d) Adaptive prediction domain: Self-adaptive prediction domain enables the vehicle to adjust the trajectories’ length according to the obstacles’ information to avoid the insolvable solution state. 2. Objective function: The multi-objective function is defined as $J(v,\omega )=-\sigma (\mu \cdot heading(v,\omega )+\beta \cdot dist_{g}(v,\omega )+\eta \cdot dist_{o}(v,\omega )+\gamma \cdot vel(v,\omega ))$ With respect to the current position, target position, and orientation of the vehicle this function trades off the following aspects: (a) Target heading: heading is a measure of progress toward the target position. It is maximal if the vehicle moves directly toward the target. (b) Target distance: $dist_{g}$ is the distance to the goal location on the end of the prediction trajectory. The smaller the distance to the goal location, the higher the vehicle’s desire to move around it. (c) Clearance: $dist_{o}$ is the minimum distance from the obstacles along the current planned trajectory. The smaller the distance between the vehicle and obstacles, the higher the safety risk of the vehicle. (d) Velocity: vel is the translational velocity of the vehicle. The parameter σ smooths the global weighted sum of the four components.

Furthermore, the vehicle’s motion is also subject to mechanical operational limits, where the steering angle of the wheels imposes restrictions on the vehicle’s lateral movement. The corresponding sampled space is as follows:

(6) $$V_{b}=\left\{(v,\omega )\left||\theta_{\mathrm{wheel}}|\leq \theta_{\mathrm{wheel}}^{\max}\right.\right\}$$

where $\theta_{\mathrm{wheel}}^{\max}$ denotes the maximum steering angle of the vehicle’s front wheels.

Additionally, it is imperative to consider the vehicle’s dynamic safety performance to mitigate the risk of vehicle rollovers stemming from excessive steering speeds during high-speed maneuvers. In accordance with the literature [23,24,25], the critical rollover prevention velocity model for the Ackermann steering model with rear-wheel drive and front axle steering is employed as:

(7) $$\begin{array}{l} V_{c}=\left\{(v,\omega )\left|v\leq v_{c}\right.\right\}\\ v_{c}=\left\{\begin{array}{cc} v_{\max} & \theta_{\mathrm{steer}}<0.005\\ \sqrt{\frac{gh_{g}}{\left|\tan\theta_{\mathrm{steer}}\right|}} & \theta_{\mathrm{steer}}\geq 0.005 \end{array}\right. \end{array}$$

where $v_{c}$ and $h_{g}$ denotes the corresponding critical rollover prevention velocity and height of gravity center, respectively.

Hence, the dynamic window $V_{r}$ can be calculated by the intersection of the above velocity sample spaces:

(8) $$V_{r}=V_{s}\cap V_{d}\cap V_{a}\cap V_{b}\cap V_{c}$$

## 5. Simulations and Discussions

It is essential to emphasize that the rapid descent speed of airborne risks places constraints on the available planning time. Therefore, this paper confines its investigation to scenarios in which the vehicle’s air–ground risk avoidance problem theoretically has feasible solutions indeed.

In the simulation, the vehicle commences its mission from a stationary position toward the designated target location. The specific vehicle parameters and simulation settings are outlined in Table 1 and Table 2, respectively. The simulation pertaining to airborne factors holds significance only when an aerial object is deemed a risk through the risk assessment method, indicating a real possibility of a collision with the vehicle. To facilitate a meaningful comparison, a comparative simulation approach is employed. Hence, in each simulation, three airborne obstacles are introduced from different directions and velocities. If the vehicle maintains its current trajectory without alterations, it will face consecutive impacts with these obstacles. At three specific time points, synchronized launches of three aerial trajectories occur, all with identical velocity and initial altitude, thus allowing for a comprehensive performance evaluation. In the comparative simulations, to demonstrate the validity of the airborne obstacles, we maintain their direction and velocity properties while adjusting corresponding initial positions, to ensure that these airborne obstacles genuinely pose a threat to the UTV in the comparative simulations. In the different simulations, the shared attributes of the three airborne obstacles are configured in Table 3. The real-time planning objective is considered achieved when the vehicle approaches within a 0.5-m radius of the target location.

**Figure 5:**
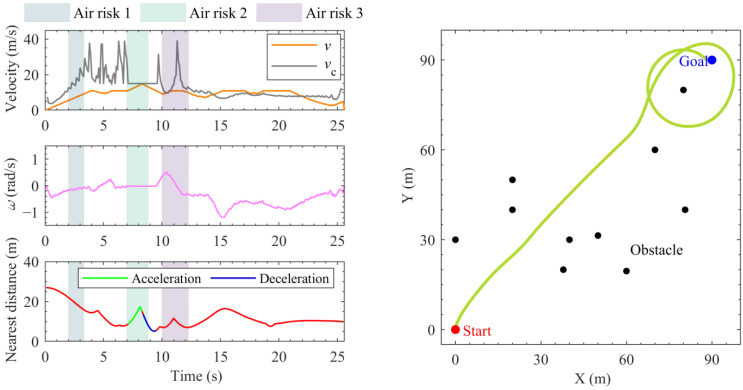
The motion planning results for the traditional DWA.

The movement settings of the given 10 obstacles are shown in Appendix A. Figure 5 presents the traditional DWA-based hierarchical local planning simulation with air–ground risks in the construction scenario. The labels “Air risk 1–3” in the figure correspond to the time window, which spans from the risk assessment to the risk disappearance (impact to the ground). The labels “Acceleration/Deceleration” in the figure correspond to the non-steering-based command of the hierarchical decision, and the rest red lines correspond to the steering-based command. It is illustrated that the traditional DWA-based planning process takes 25.6 s, but results in a continuous exceeding toward the rollover critical velocity $v_{c}$. Moreover, influenced by the nearby obstacles and mechanical constraints, the UTV ultimately follows a much redundant trajectory to the goal destination. As a result, this approach is deemed unsuccessful and ineffective.

**Figure 6:**
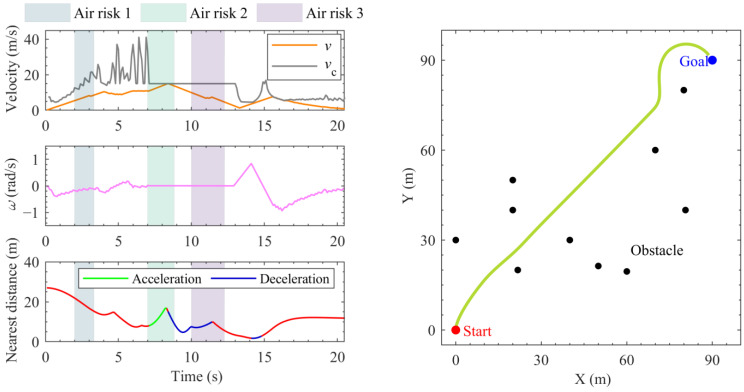
The motion planning results for the proposed ADWA.

Figure 6 presents the proposed ADWA-based hierarchical local planning simulation with air–ground risks in the construction scenario. The designed adaptive prediction horizon is set as:

(9) $$t_{p}=\tau t_{\mathrm{pm}}\;\;\tau =clip\left\{\frac{1.0}{n_{o}+\zeta },[0.4,1.0]\right\}$$

where $t_{\mathrm{pm}}$ denotes the max prediction time, set to 1.5 s in this paper. $n_{o}$ denotes the number of obstacles around within 20 m. ζ is a positive value to prevent the calculation error when $n_{o}=0$, set to one. clip denotes the boundary-constraining function. Thus, when there are excessive obstacles near the vehicle, the system will automatically reduce the prediction horizon to prevent unsolvable scenarios resulting from the over-large prediction scale. And the calculation cost can be correspondingly reduced.

Throughout the planning process, hierarchical planning decision operations are executed, involving obstacle avoidance maneuvers achieved through pure acceleration and deceleration. This planning effect illustrates the necessity and effectiveness of layered control. It is depicted that the proposed ADWA-based planning process takes 20.9 s, in which the vehicle’s velocity remains consistently below the rollover critical velocity $v_{c}$. The distance from the UTV to the real-time nearest obstacle consistently adheres to the designed safety distance threshold. Additionally, in the final phase of planning around the goal destination, the corresponding planning trajectory still performs a smooth tendency. This overall planning process reflects that the proposed ADWA exhibits clear advantages in terms of safety and feasibility compared to the traditional DWA.

**Figure 7:**
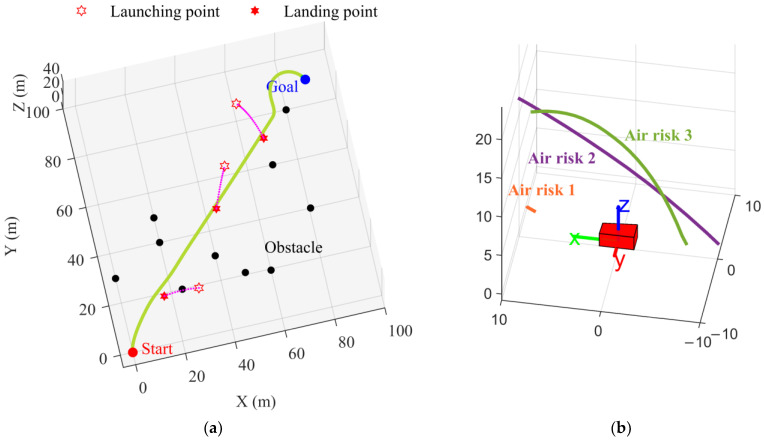
The motion planning results for the proposed ADWA: (**a**) Aerial view; (**b**) Local view.

The corresponding aerial view is illustrated in Figure 7. It shows that the landing point of the airborne risk intersects with the trajectory, which seems to be a collision. In fact, combining Figure 6 and Figure 7b, the UTV adopts a series-coupled acceleration/deceleration and steering maneuver to circumvent the risk presented by airborne risk 1. This adjustment effectively redirected the initial collision trajectory of risk 1 to the vehicle’s lateral side. Similar avoidance strategies were employed to evade risks 2 and 3. The simulation results demonstrate the effectiveness of the hierarchical decision mechanism and the proposed ADWA method for the air–ground risk in construction scenarios.

## 6. Conclusions

In this paper, we have demonstrated a novel integrated autonomous dynamic navigation approach for construction scenarios with composite air–ground risks. In response to the potential falling obstacle risk of the construction scene, a hierarchical self-adaptive DWA algorithm has been accordingly proposed. Through the hierarchical decision mechanism, the vehicle is directed to minimize air–ground risks through non-steering maneuvers. In the proposed ADWA algorithm, the self-adaptive prediction horizon and the rollover critical velocity window were proposed, enabling the vehicle to fine-tune planning windows in high-risk scenarios while ensuring planning safety. Simulation results show that the proposed hierarchical self-adaptive dynamic window approach demonstrates higher planning efficiency and safety toward the traditional dynamic window approach, with the UTV consistently staying within the safety constraints of the construction scenario. This work provides an efficient solution for vehicle motion planning under air–ground-coupled risks. However, there is still enhancement potential in the algorithm’s adaptive capability. Future work will focus on improving algorithm performance and conducting relevant experimental validation.

## Figures and Tables

**Table 1:** Physical parameters of the UTV.

Parameter	$v_{\max}$ (m)	$\omega_{\max}$ (deg/s)	$a_{\max}$ (m/s^2^)	$\alpha_{\max}$ (deg/s^2^)	$\theta_{\mathrm{wheel}}^{\max}$ (deg)	Body Size (m)	Mass Center Height (m)	Wheelbase (m)
Value	15.0	60.0	3.2	60.0	30.0	3.5 × 2.0 × 1.6	1.0	2.0

**Table 2:** Simulation settings.

Parameter	Initial Location (m)	Target Location (m)	Sample Frequency (Hz)	Perception Distance (m)	Safe Radius (m)	Air Resistance Coefficient	Air Density (kg/m^3^)
Value	(0.0, 0.0)	(90, 90)	10	30.0	3.0	0.2	1.2

**Table 3:** Shared attributes of the airborne risks.

Parameter	Initial Height (m)	Initial Velocity Vector (m/s)	Mass (kg)	Launching Time (s)
risk 1	15.0	(−10.0, 3.0, −5.0)	2.0	2.0
risk 2	25.0	(−3.0, −5.0, −5.0)	7.0	7.0
risk 3	25.0	(4.0, −4.0, 0.0)	10.0	10.0

## Data Availability

The data that support the findings of this study are available from the corresponding author upon reasonable request.

## Appendix A

**Table A1:** Initial obstacle information.

Obstacle ID	Initial Location (m)	$v_{x}^{o}(0)$ (m/s)	$v_{y}^{o}(0)$ (m/s)
1	(0, 30)	0.0	0.0
2	(60, 20)	7.0	0.0
3	(50, 50)	0.0	5.5
4	(20, 40)	0.0	1.0
5	(20, 50)	0.0	1.0
6	(60, 20)	0.0	−8.0
7	(70, 40)	10	0
8	(40, 30)	0	0
9	(70, 60)	0	0
10	(80, 80)	0	0

In Table A1, $v_{x}^{o}(0)$ and $v_{y}^{o}(0)$ denote the initial horizontal and longitudinal velocity of the obstacles, respectively. Once the motion of obstacles extends beyond the rectangular boundary that originated from the coordinates (20, 20) to (80, 80), the subsequent speed will be set to:

(A1) $$\left\{\begin{array}{l} v_{x}^{o}(t)=-0.7v_{x}^{o}(0)\\ v_{y}^{o}(t)=-0.7v_{y}^{o}(0) \end{array}\right.$$
